# Exploring supervised and unsupervised methods to detect topics in biomedical text

**DOI:** 10.1186/1471-2105-7-140

**Published:** 2006-03-16

**Authors:** Minsuk Lee, Weiqing Wang, Hong Yu

**Affiliations:** 1 Department of Biomedical Informatics, Columbia University, 622West, 168th Street, VC-5, NY 10032, USA; 2 Department of Molecular Pharmacology, Albert Einstein College of Medicine, Bronx, NY 10461, USA

## Abstract

**Background:**

Topic detection is a task that automatically identifies topics (e.g., "biochemistry" and "protein structure") in scientific articles based on information content. Topic detection will benefit many other natural language processing tasks including information retrieval, text summarization and question answering; and is a necessary step towards the building of an information system that provides an efficient way for biologists to seek information from an ocean of literature.

**Results:**

We have explored the methods of *Topic Spotting*, a task of text categorization that applies the supervised machine-learning technique naïve Bayes to assign automatically a document into one or more predefined topics; and *Topic Clustering*, which apply unsupervised hierarchical clustering algorithms to aggregate documents into clusters such that each cluster represents a topic. We have applied our methods to detect topics of more than fifteen thousand of articles that represent over sixteen thousand entries in the Online Mendelian Inheritance in Man (OMIM) database. We have explored bag of words as the features. Additionally, we have explored semantic features; namely, the Medical Subject Headings (MeSH) that are assigned to the MEDLINE records, and the Unified Medical Language System (UMLS) semantic types that correspond to the MeSH terms, in addition to bag of words, to facilitate the tasks of topic detection. Our results indicate that incorporating the MeSH terms and the UMLS semantic types as additional features enhances the performance of topic detection and the naïve Bayes has the highest accuracy, 66.4%, for predicting the topic of an OMIM article as one of the total twenty-five topics.

**Conclusion:**

Our results indicate that the supervised topic spotting methods outperformed the unsupervised topic clustering; on the other hand, the unsupervised topic clustering methods have the advantages of being robust and applicable in real world settings.

## Background

*Topic detection *is defined in this application as a task that automatically identifies topics (e.g., "Gene Function" and "Biochemical Features") based on the information content of a scientific article. Topic detection is an important field that can benefit many other natural language processing tasks including information retrieval, summarization, and question answering. Information retrieval may organize the retrieved results into topics to facilitate user navigation (e.g., (Hearst & Pedersen 1996)); for example, molecular biologists may be interested in browsing articles related to "gene function"; and structural biologists may prefer to browse the articles related to "protein structure". Knowing the topics of relevant articles will assist human annotators to create knowledge bases efficiently. For example, T1Dbase [1] is a database designated to genes related to Type I diabetes mellitus. The annotators need to identify literature articles that are relevant to specific genes related to Type I diabetes mellitus with the topic of "cellular molecular biology and genetics". Topic detection is crucial for the *summarization*, the technique that condenses information while preserving information content. A summarization system may summarize documents within a topic and aggregate information across topics. Topic detection is also important for question answering, the techniques that produce short texts to answer users' specific questions. For example, Yu and Hatzivassiloglou [2] made extensive uses of topic detection techniques to extract specific answers

Research in topic detection has started since 1998 as a part of Topic Detection and Tracking (TDT) under the DARPA Translingual Information Detection, Extraction, and Summarization (TIDES) program. The closely related task is text categorization that has become very standard to organize text [3]. On the other hand, there is much less reported work in the biomedical domain. A closely related work is by Wilbur [4] who developed an EM algorithm to identify "theme" or topics from a large collection of text. However, his method has not yet been evaluated in the tasks of topic detection. Another related work is by Hearst [5] who has built an information retrieval system in which retrieved PubMed citations are aggregated based on the Medical Subject Headings (MeSH) that were assigned to the articles. MeSH terms in this case serve as the topics of the citation. This approach benefits from the fact that the MeSH terms represent the content of the full-text articles of the PubMed citations and each MEDLINE citation has assigned MeSH terms by the NLM annotators. On the other hand, there are several disadvantages of this approach. Since each MEDLINE abstract has an average of eleven MeSH terms assigned and therefore it might result in too many topics for a collection of PubMed citations; many MeSH terms are also too specific (e.g., gene or protein names) to be used as topics. In addition, the MeSH terms are related hierarchically, and frequently both the parent and the child are assigned to the same citation; this results in "redundant" topics. Much of other related work focus on the tasks of associating genes to Gene Ontology codes [6-8].

This work presents the first attempt that applies both *topic spotting *and *topic clustering*, two topic detection methods that automatically detect topics in the biomedical text. We evaluate our methods to detect topics from more than fifteen thousand reference articles that are cited over sixteen thousand entries in the Online Mendelian Inheritance in Man (OMIM) database [9]. Additionally, we have evaluated our methods to detect topics that are annotated by biologists. We have explored the semantic features, which come from the biomedical, domain-specific knowledge resources; namely, the Medical Subject Headings (MeSH) and the Unified Medical Language System (UMLS), to facilitate the task of topic detection. We have found that the semantic features enhance the performance of topic detection in the biomedical texts.

## Results

We report the results of applying the models of topic spotting and topic clustering to detect topics of 15,405 articles that have been incorporated into the OMIM database, as well as an additional set of 56 articles that are cited in four biological review articles.

### OMIM statistics

**Figure 1:**
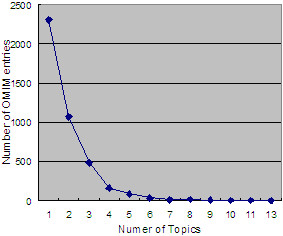
The number of the OMIM entries as a function of the number of topics.

Figure 1 plots the number of the OMIM entries as the function of the number of topics per entry. As described in the Methods section, each OMIM entry incorporates zero, one, two, or more topics. The OMIM incorporate references under each topic. The results show that 2,062 OMIM entries have only one topic and 1,161 OMIM entries have two topics. The number of OMIM entries decreases when the number of topics increases and it follows the power law distribution *y *= 2980.95*e^-0.64*x^, where x represents the number of topics and y represents the number of OMIM entries. There are only two OMIM entries that incorporate the highest 13 topics.

### Topic spotting

Table 1 reports the accuracy of topic spotting in the OMIM database when applying the naïve Bayes machine-learning classifier. The accuracy is defined as the percentage of documents with the correct topic predictions by the naïve Bayes classifier. We have reported results of applying different combinations of features; including the combination of word features in title and abstract, and the additional semantic features (i.e., the MeSH terms and the UMLS semantic type).

Our results show that the classification are in general enhanced when we include the MeSH terms and the UMLS semantic types as the additional features and the best performance is achieved by combining all four features; namely, semantic types, MeSH terms, and the words in both title and abstract; this leads to a 66.4% accuracy. As described in the Methods section, the MeSH terms are assigned by the National Library of Medicine annotators to each MEDLINE article. The UMLS has assigned semantic types to each MeSH term. Note that a baseline system that classifies every document into the largest category (i.e., *CLINICAL FEATURES*) would achieve an overall accuracy of 19.8% and random guessing would lead to an overall accuracy of 4%; and therefore the topic detection method with naïve Bayes is significantly better than the random guessing. When we reduced the total number of topics to the top ten topics, we have obtained the best accuracy to be 72.9% with a baseline of 21.2%.

Our results show that the classification performance, on the other hand, significantly decreases (a drop of 15.4%) when we replace the MeSH terms with their general categories specified in the MeSH hierarchy; the performance also slightly decreases when we add in the general MeSH terms in addition to the original assigned MeSH terms. Our results also show that using the semantic types alone as features also have disappointing performance (i.e., 54.4%), although the performance is notably higher than using the general categories of the MeSH terms (i.e., 47.4%).

### Topic clustering

**Figure 2:**
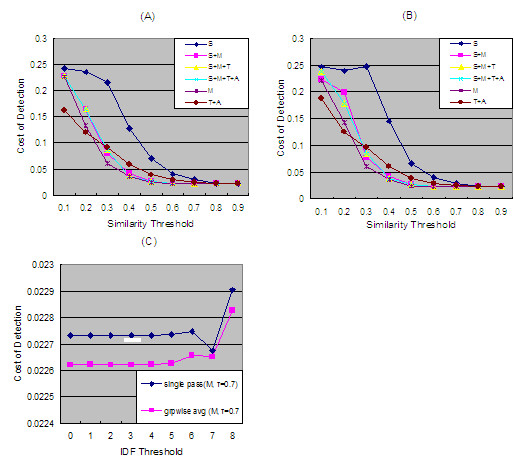
**Topic clustering in OMIM**. The cost of detection of *Single-pass *(A) and *Group-wise-average *(B) with different features; namely, semantic types only (S); combined semantic types and MeSH terms (S+M); semantic types with MeSH terms and title (S+M+T); all four features (S+M+T+A); the MeSH terms alone (M); and combined title and abstract (T+A). (C) Comparison of the cost of detection between single-pass and group-wise-average with MeSH terms alone as features and similarity threshold τ = 0.5.

**Figure 3:**
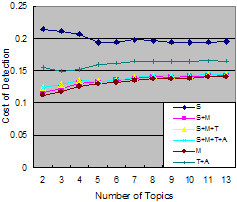
Topic Clustering as a function of number of topics (similarity of threshold τ = 0.5.).

Figure 2, 3, 4 report the results of topic clustering that have been evaluated in the OMIM database and the additional set of four biological review articles. Note that topic clustering only clusters similar documents and does not create topic terms for each document cluster. Our evaluation of topic clustering based on the OMIM database is therefore an approximation in which we assume that each document cluster generated by the topic clustering corresponds to each topic indicated by the OMIM database.

Recall that we have applied both the group-wise-average and single-pass hierarchical clustering algorithms for topic clustering. Since the previous results of topic spotting (shown in Table 1) have shown that the general MeSH categories are not useful features for topic detection, we report only the results with the feature combinations including the semantic types, the MeSH terms, and the bag-of-words in title and abstract.

We report *cost of detection *[10] as the evaluation metrics for topic clustering. Cost of detection is defined in the Evaluation Metrics of the Methods section. Figure 2(A) and 2(B) report the results of cost of detection with the single-pass algorithms and the group-wise average respectively as a function of similarity threshold (0.1~0.9) in different feature sets. Both single-pass and group-wise-average hierarchical clustering algorithms are described in the Methods section. In both cases, the higher the threshold, the lower cost of detection or the better the system. The results show that the best system (with the lowest cost of detection value) is group-wise-average with MeSH terms alone at the similarity threshold τ = 0.7 to achieve 0.0226 (*P*_*M *_= 58.9%, *P*_*FA *_= 2.12e-2%), which is comparable with the single pass's performance 0.0227 (*P*_*M *_= 59.0%, *P*_*FA *_= 3.03e-2%). *P*_*M *_is defined as the probability of missing an article in a topic and *P*_*FA *_is defined as the probability of assigning an article to wrong topic.

Since previous studies have shown that feature selections have an impact on the performance of topic detection [11], we have therefore explored feature selection by the Inverse Document Frequency (IDF) threshold. IDF is defined as log⁡2(Nn)
 MathType@MTEF@5@5@+=feaafiart1ev1aaatCvAUfKttLearuWrP9MDH5MBPbIqV92AaeXatLxBI9gBaebbnrfifHhDYfgasaacH8akY=wiFfYdH8Gipec8Eeeu0xXdbba9frFj0=OqFfea0dXdd9vqai=hGuQ8kuc9pgc9s8qqaq=dirpe0xb9q8qiLsFr0=vr0=vr0dc8meaabaqaciaacaGaaeqabaqabeGadaaakeaacyGGSbaBcqGGVbWBcqGGNbWzdaWgaaWcbaGaeGOmaidabeaakmaabmaabaWaaSaaaeaacqWGobGtaeaacqWGUbGBaaaacaGLOaGaayzkaaaaaa@3615@ where N is the number of documents in the text collection and n is the number of documents containing this feature in the text collection. We have performed IDF feature selections based on both single pass and group-wise-average. The results (Figure 2(C)) show that the IDF threshold yields an improvement over the cost of detection. However this improvement comes at the cost of higher false alarm (or false positive) as noted. The lowest cost of detection is when IDF threshold = 7, which has the cost of detection of 0.02268 (*P*_*M *_= 52.0%, *P*_*FA *_= 4.99e-1%); this corresponds to an accuracy of 34.7%. The results also show that the differences between the single-pass and group-wise average further narrowed.

In our hierarchical clustering algorithm, a higher similarity threshold indicates a greater number of clusters will be generated by the algorithm; this will generally lead to a greater *P*_*M *_and lower *P*_*FA*._. However, the number of clusters generated might be too many or too few for a given threshold and the performance might not be consistent over varied numbers of topics. We have therefore examined the topic clustering performance in OMIM as a function of topics for our best system (features include the MeSH terms only, τ = 0.7). As shown in Figure 3, the performance of single-pass slightly decreases as the number of topics increases.

**Figure 4:**
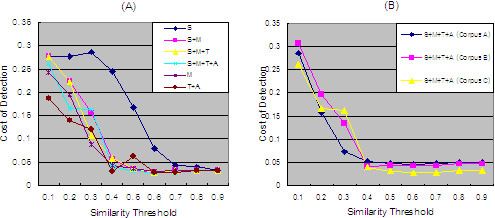
**Topic Clustering of articles cited in biological review articles**. (A) The cost of detection of Group C with different features; namely, semantic types only (S); combined semantic types and MeSH terms (S+M); semantic types with MeSH terms and title (S+M+T); all four features (S+M+T+A); the MeSH terms alone (M); and combined title and abstract (T+A). (B) The cost of detection of three groups (A, B and C) with the semantic types as the feature.

Figure 4 shows the results of applying the topic-clustering models to detect a total of 14 topics of an additional set of articles that are cited in the total of four biological review articles. We report the results of detecting the total 56 articles that are cited (denoted at *Group A*), 44 articles when we removed the "review" articles (denoted as *Group B*) and 27 articles when the biologist (Dr. Wang) has further selected the most relevant articles (denoted as *Group C*); the results show that the cost of detection decrease in the same order. The best feature for Group A and B is the combination of semantic types and MeSH terms with cost of detection 0.042 (*P*_*M *_= 58.81%, *P*_*FA *_= 0%, Accuracy = 32.3%), and 0.038 (*P*_*M *_= 53.56%, *P*_*FA *_= 0%, Accuracy = 36.5%) when similarity threshold is τ = 0.5. The best feature for Group C is the combination of words in title and abstract, semantic types, and MeSH terms with cost of detection 0.027 (*P*_*M *_= 39.88%, *P*_*FA *_= 0%, Accuracy = 50.0%) with similarity threshold of τ = 0.6.

## Discussion

Our results show that naïve Bayes can achieve the highest accuracy (66.4%) to automatically detect the topic of 15,405 OMIM article to belong to the categories of 25 topics specified by the OMIM; which is significantly higher than the baseline system (19.8%). The results suggest that supervised machine-learning may be used to assist the manual efforts of OMIM annotation, in addition to the general NLP benefits that we have described in Introduction.

We have found that the UMLS semantic types consistently enhance the performance of topic detection when used in addition to other features (e.g., bag-of-words). This is not surprising because we have found that the UMLS semantic network provide a comprehensive coverage of a high-level conceptual knowledge [12].

Our results also show that the MeSH terms are useful features as they have enhanced the performance of topic detection. On the other hand, we have found a drop in performance when we replace the MeSH terms with their general terms; this might be partially caused by the fact that the MeSH terms are organized in strict hierarchy and therefore result in fewer useful features compared with the multiple semantic types. For example, the MeSH term *Protein Tyrosine Phosphatase *corresponds to only one second-level general MeSH term *Enzyme*, while the same term has two UMLS semantic types assigned (i.e., *Enzyme *and *Amino Acid, Peptide, or Protein*). Our results suggest that the approach of directly applying the MeSH terms as topics might not be optimal for assigning topics to biomedical texts, particularly in the OMIM database.

Although topic spotting has the best performance, it requires a large set of predefined topics with an available training corpus. Because the training corpora, in most of cases, are very difficult to obtain, topic spotting has a significant limitation. In fact, the field of biology is very dynamic and biologists frequently organize different collections of literature articles based on different biological purposes; this is reflected by the fact that different biological review articles incorporate different collections of articles under different topics created by the authors of the review articles.

The unsupervised topic clustering methods have the advantages of not depending on a training corpus and therefore being robust and being likely to be applicable to real world settings. Since unsupervised approaches in general under-perform supervised ones, it is not surprising that our results have shown that the best topic clustering algorithms have achieved a lower accuracy (i.e., 34.6%), when comparing to the accuracy of topic spotting of an accuracy of 66.4%, to automatically detect topics of 15,405 cited articles in the OMIM. On the other hand, the performance of topic clustering is significantly higher (i.e., more than 14%) than the baseline of 19.8% that classifies every document into the largest category and more than 30% higher than the random guessing 4%. Additionally, our results show that the topic clustering model performs reasonably well with our additional annotated corpus of a total of 57 articles that are cited in four review articles; the performance of the topic clustering increases with the quality of this corpus, and achieves the highest of 50% accuracy. Topic clustering therefore provides an alternative, robust unsupervised approach that could cluster documents and detect topics dynamically.

We have applied two approaches for topic clustering; namely, single-pass and group-wise-average. Our evaluation results show that group-wise-average out-performed single-pass; however, the differences are subtle (~0.0001 in cost of detection). Because it is computationally much more expensive to apply the group-wise-average approach in a large text collection, we conclude that single-pass is an excellent alternative for topic-clustering in a large collection of text.

One disadvantage of the topic clustering methods is that the method only aggregates documents into clusters and does not provide a topic term for each cluster. To automatically identify a topic term, we may apply the work of [13] to automatically obtain keywords from each cluster and apply the keywords as the topics to represent the cluster. We may also apply the work of [4] to identify "theme" words to be used as the topics.

Another disadvantage of our study is that we have evaluated both topic spotting and topic clustering methods to detect documents that incorporate only one topic, not multiple topics. However, in reality, it is frequent that a document incorporates multiple topics. To detect multiple topics, we may apply topic spotting with binary classification (i.e., for each topic, assigns yes or no to the document) so that a document can be assigned multiple topics. To apply topic clustering methods for the purpose of detecting multiple topics, we may first apply [4] to identify a list of the "theme" features for each potential topic. Given a document, we measure document similarity with the "theme" features for each topic and then assign yes or no to the document for the topic.

Our results show that the lowest value of cost of detection or the best system for topic clustering is 0.0226 (*P*_*M *_= 58.9%, *P*_*FA *_= 2.12e-2%), which is higher than the reported cost of detection (~0.005) by the systems that apply hierarchical clustering algorithms to detect topics in news articles [11]. The difference may be explained by the quality of the evaluation data. The TDT2 collection has been annotated by specialists for the purpose of evaluating topic detection and therefore has a high quality and the topics in the TDT2 collection are quite distinct. The biological texts we have experimented with (i.e., OMIM and the additional set of biological review articles) are related by the same genes with related the biological topics (e.g., "Cloning" and "Gene Function") and therefore it is harder to separate biological texts for the purpose of topic detection. In addition, our results have shown that the performance of topic clustering is enhanced significantly when the quality of the evaluation corpus increases. In this case, the accuracy increases to 50.0% from 32.3% when a biologist has manually excluded the articles that do not belong to the assigned topics. All our evaluation results of topic clustering have achieved significantly above the baselines, and therefore the results suggest that topic clustering methods are applicable to biomedical text, although there is still a big room to enhance the performance to make the methods real useful.

## Future Work

To enhance the performance, one may further expand feature selection to biological functionally important words. For example, "phosphorylation" and "3-D" are important word which might sufficiently separate "protein function" from "protein structure".

## Conclusion

This study represents the first and the state-of-the-art topic detection methods in biomedical texts. The evaluation has concluded that the supervised topic spotting has the highest performance for topic detection in the OMIM data. Our results show that although the unsupervised topic clustering methods under-perform than the topic spotting methods, the performance are significantly above the baseline. Additionally, the performance of the topic clustering methods is enhanced when applying to detecting topics that are defined by biologists in their review articles. Our results show that topic clustering methods are robust to deal with real world events. The results also conclude that the performance of topic clustering increases with the quality of data.

## Methods

We have built two statistical machine-learning models; namely, *topic spotting *and *topic clustering*, to automatically detect topics in biomedical texts. We have applied the two models to detect a total of 25 topics of more than 15,000 articles that were cited in the biological database the Online Mendelian Inheritance in Man (OMIM), and a total of 14 topics of 56 additional articles that are cited in four biological review articles. In the following, we will first describe the OMIM database and the additional text database we used to evaluate the two models. We will then describe the topic spotting and clustering models.

### Text collection: OMIM

The OMIM database is an expert-annotated database that organizes genes and genetic disorders [9]. Each OMIM entry has a full-text summary of a genetically determined phenotype and/or gene. The entries are organized into different topics. Each topic presents a text summary along with literature references that lead to the summary. For example, querying the OMIM with the gene "PTEN" results in literature reports that are grouped into topics including *CLONING, GENE FUNCTION, BIOCHEMICAL FEATURES, MAPPING, MOLECULAR GENETICS*, and *ANIMAL MODEL*. Under each topic, the OMIM entry incorporates references articles. Those references articles have been used as both the training set and the gold standard to evaluate our topic-detection task.

Currently, the OMIM incorporates a total of 107,632 unique articles as references to describe 16,752 entries. We found that many of the references were cited in the general description sections of the OMIM entries and did not have specific topics assigned. For example, the OMIM entry for "Adenylosuccinate Synthetase" (OMIM ID = 103060) incorporates sections including the general description, and other topic sections (e.g., Mapping). The general description cites five reference articles that do not have specific topic assigned. After removing those non-topic-specific references, we obtained a total of 36,772 unique articles that have assigned by OMIM with one or more topics. We further excluded the references articles that have two or more topics assigned and have included the non-redundant reference articles (a total of 20,644). Furthermore, among 20,644 reference articles, we have only included 15,405 articles that also appear in our MEDLINE database (NLM licensing 1966–2004). Table 2 lists all the topics that appear in the OMIM with the number of literature articles that have been assigned to each topic for the tasks of topic detection.

The OMIM provides excellent large number of the training sets that are necessary for the applications of topic spotting; the OMIM data can also be used to evaluate the unsupervised topic clustering model. However, we cannot directly use the OMIM data as they are because the OMIM entry provides only reference citations (i.e., the standard citation that incorporates the authors, the title, the journal name, and publication date), and do not provide the content or the abstracts of the reference articles. It is those content or the abstracts from which we can extract features to apply our topic detection models. We have therefore developed a program to automatically map with 100% precision the OMIM reference citations to the PubMed citations (e.g., title, abstract, and the assigned Medical Subject Headings or the MeSH terms).

### Text collection: Biology review articles

The OMIM is a highly specialized database that focuses on genetic disorders. To test the generality of our models of topic detection, we have randomly selected four review articles from Cell (3) and Accounts of Chemical Research (1), two leading biological journals that focus on different biological sub-domains. Each review article incorporates topics assigned by the writer(s); literature references are specified under each topic. For example, in (Warner 2001) the author has described three topics: 1) The RNAs of Ribosome Formation; 2) The Proteins of Ribosome Formation; and 3) The Ribonucleoproteins of Ribosome Formation. Under each topic, the author has cited articles to support the descriptions. We have manually downloaded the title, abstract, and the MeSH terms of each cited reference.

One of the co-authors (i.e., Dr. Wang) is an expert in biology; she found that not all cited articles are relevant to the topics. A notable fact is that many cited articles are review articles. Obviously, review articles must cover multiple topics and therefore might not be the most representative articles for a specific topic. Dr. Wang further discovered that some of the cited non-review articles are not necessarily directly related to the assigned topics. For example, the article (Rigaut et al 1999) is one of the three cited article under the topic "the Ribonucleoproteins of Ribosome Formation". However, this article only describes a general technique, and does not provide specific content that is directly related to the topic. Dr. Wang has therefore manually examined all cited articles in those review articles and selected the ones that she thought were definitely relevant. Note that Dr. Wang annotated the data independently and she did not participate in the tasks of topic detection. We have applied the topic clustering methods to detect topics for each set of the documents and reported the average performance.

#### Model 1: Topic spotting

Topic Spotting is a task of document categorization: Given a set of n predefined topics, the task is to determine the topics present in each document. We have applied the supervised machine-learning approach naïve Bayes, which has been used successfully for text categorization tasks (Sebastiani 2002).

***Naïve Bayes ***is commonly used in machine learning and text categorization. Naïve Bayes is based on Bayes' Law and assumes conditional independence of features. For text categorization, this "naive" assumption amount to the assumption that the probability of seeing one word in a document is independent of the probability of seeing any other word in a document, given a specific category. Although this is clearly not true in reality, naive Bayes has been useful for many text classification and other information retrieval tasks.

#### Model 2: Topic clustering

Topic spotting applies to the case in which topics are pre-defined. In reality, it may be hard to pre-define topics. For example, in the tasks of information retrieval and question answering, the topics are ad-hoc depending on the text collection to be analyzed. Topic clustering provides a robust alternative that does not require the topics to be predefined. Specifically, topic clustering applies unsupervised hierarchical clustering algorithms to automatically group documents into different topics based on the similarity among the documents to be analyzed.

The central theme of topic clustering is the hierarchical clustering algorithms, which are well-established algorithms that are widely used in many areas including biological sequence alignment [14] and gene expression analyses [15]. In this application, hierarchical clustering groups a collection of texts into subsets or "clusters", such that those within each cluster are more closely related to one another than texts assigned to different clusters; each cluster represents a specific topic that is different from other clusters. Topic clustering is a key component of the TDT research. Hatzivassiloglou and colleagues [11] have compared several commonly used hierarchical clustering algorithms and concluded that *Group-Wise Average *performs the best, while *Single-Pass Clustering *has the advantage of being the fastest. In the biomedical domain, frequently there is a need to analyze a large number of texts. For example, querying PubMed about the drug *aspirin *results in over thirty thousand MEDLINE records. A faster algorithm may therefore still be valuable in the real applications at the cost of a lower performance, if the tradeoff is not significant. In this study, we have evaluated both algorithms for topic clustering in biomedical texts. Both algorithms measure pair-wise document similarity based on the vector space model [16] that is typically applied in information retrieval. We compute cosine (TF*IDF) similarity. TF refers to "term frequency" in the given document and IDF refers to "inverse document frequency". IDF is defined as Log2(Nn)
 MathType@MTEF@5@5@+=feaafiart1ev1aaatCvAUfKttLearuWrP9MDH5MBPbIqV92AaeXatLxBI9gBaebbnrfifHhDYfgasaacH8akY=wiFfYdH8Gipec8Eeeu0xXdbba9frFj0=OqFfea0dXdd9vqai=hGuQ8kuc9pgc9s8qqaq=dirpe0xb9q8qiLsFr0=vr0=vr0dc8meaabaqaciaacaGaaeqabaqabeGadaaakeaacqWGmbatcqWGVbWBcqWGNbWzdaWgaaWcbaGaeGOmaidabeaakmaabmaabaWaaSaaaeaacqWGobGtaeaacqWGUbGBaaaacaGLOaGaayzkaaaaaa@35D6@ where N is the number of documents in the text collection and n is the number of documents containing this word in the text collection. TF gives a measure of the importance of a term within the particular document and IDF is a measure of the general importance of the term.

**Group-Wise Average **starts with the entire set of documents. It identifies pair-wise document similarity based on cosine TF*IDF similarity. It then merges the two documents with the highest similarity into one cluster. It then re-evaluates pairs of documents/clusters; two clusters are merged if the average similarity across all pairs of documents within the two clusters is equal to or greater than a predefined threshold. In the event of multiple clusters that can be merged at any time, the pair of clusters with the highest similarity is always preferred. For computational complexity, we can analyze the algorithm in terms of number of the pairs for which it needs to compute the similarity. To facilitate our analysis, we break the clustering into first pass and rest of the passes. During the first pass, first document requires no computation, second document requires similarity computation against the first, and third document requires similarity computation against first and second. Evidently, as the pattern shows, first needs to compute the similarity ∑k=1N−1k=12*N*(N−1)
 MathType@MTEF@5@5@+=feaafiart1ev1aaatCvAUfKttLearuWrP9MDH5MBPbIqV92AaeXatLxBI9gBaebbnrfifHhDYfgasaacH8akY=wiFfYdH8Gipec8Eeeu0xXdbba9frFj0=OqFfea0dXdd9vqai=hGuQ8kuc9pgc9s8qqaq=dirpe0xb9q8qiLsFr0=vr0=vr0dc8meaabaqaciaacaGaaeqabaqabeGadaaakeaadaaeWbqaaiabdUgaRjabg2da9maalaaabaGaeGymaedabaGaeGOmaidaaaWcbaGaem4AaSMaeyypa0JaeGymaedabaGaemOta4KaeyOeI0IaeGymaedaniabggHiLdGccqGGQaGkcqWGobGtcqGGQaGkcqGGOaakcqWGobGtcqGHsislcqaIXaqmcqGGPaqkaaa@4137@ times. In each of the pass that follows, it needs to examine the similarity for each pair of N documents which leads to *N*^2^. Hence the overall computational complexity for group-wise average is

12*N*(N−1)+C*N2     (1)
 MathType@MTEF@5@5@+=feaafiart1ev1aaatCvAUfKttLearuWrP9MDH5MBPbIqV92AaeXatLxBI9gBaebbnrfifHhDYfgasaacH8akY=wiFfYdH8Gipec8Eeeu0xXdbba9frFj0=OqFfea0dXdd9vqai=hGuQ8kuc9pgc9s8qqaq=dirpe0xb9q8qiLsFr0=vr0=vr0dc8meaabaqaciaacaGaaeqabaqabeGadaaakeaadaWcaaqaaiabigdaXaqaaiabikdaYaaacqGGQaGkcqWGobGtcqGGQaGkcqGGOaakcqWGobGtcqGHsislcqaIXaqmcqGGPaqkcqGHRaWkcqWGdbWqcqGGQaGkcqWGobGtdaahaaWcbeqaaiabikdaYaaakiaaxMaacaWLjaWaaeWaaeaacqaIXaqmaiaawIcacaGLPaaaaaa@3F07@

where N is the number of documents to be clustered and C is the number of passes made after the first pass until no more clusters are can be merged. Note that C can take on any values from 1 in the best case to N in the worst case; this leads to the worst computational complexity to be *O*(*N*^3^).

**Single-Pass Clustering **starts with one document randomly selected from the entire set of documents. When adding in the second document, it calculates the similarity between the two documents and clusters the two documents if the similarity is above a predefined threshold. If there is more than one document or cluster of which similarity exceeds the threshold, the cluster with the highest similarity is chosen. Then it continues to add in additional documents. When an add-in document is compared with a cluster containing several documents, the similarity is the average similarity of the add-in with all of the documents in the cluster. Single-pass clustering makes a decision for each document as soon as the document is first judged, and therefore the process of clustering the entire document set is faster than with group-wise average. The computational complexity for single pass, by the same analysis of group-wise average first pass, is

12*N*(N−1)     (2)
 MathType@MTEF@5@5@+=feaafiart1ev1aaatCvAUfKttLearuWrP9MDH5MBPbIqV92AaeXatLxBI9gBaebbnrfifHhDYfgasaacH8akY=wiFfYdH8Gipec8Eeeu0xXdbba9frFj0=OqFfea0dXdd9vqai=hGuQ8kuc9pgc9s8qqaq=dirpe0xb9q8qiLsFr0=vr0=vr0dc8meaabaqaciaacaGaaeqabaqabeGadaaakeaadaWcaaqaaiabigdaXaqaaiabikdaYaaacqGGQaGkcqWGobGtcqGGQaGkcqGGOaakcqWGobGtcqGHsislcqaIXaqmcqGGPaqkcaWLjaGaaCzcamaabmaabaGaeGOmaidacaGLOaGaayzkaaaaaa@39EE@

where N is the number of documents to be clustered. Note that the computational complexity of single-pass clustering is *O*(*N*^2^), which is significant lower than the group-wise-average.

### Semantic features

For the tasks of topic spotting and clustering, in addition to bag-of-words, we have applied semantic features; namely, the Medical Subject Headings and the Unified Medical Language System concepts and semantic types.

**The Medical Subject Headings (MeSH) **is the National Library of Medicine's controlled vocabulary thesaurus that is used to index MEDLINE citations. MeSH descriptors are arranged in a hierarchical structure. The general level of the hierarchical structure includes broad headings such as "Diseases" or "Biological Sciences". More specific headings are found at lower levels of the eleven-level hierarchy; e.g. "Erysipeloid" and "Immunohistocytochemistry". There are 22,997 descriptors in MeSH. In addition to these headings, there are more than 151,000 headings called Supplementary Concept Records (formerly called Supplementary Chemical Records) within a separate thesaurus.

**The Unified Medical Language System (UMLS) **is the largest biomedical knowledge source that provides standardized biomedical concept relations and synonyms; it is maintained by the National Library of Medicine. The UMLS consists of three knowledge sources; namely, the Metathesaurus (MT), the Specialist Lexicons, and the Semantic Network (SN). The UMLS MT (2004AC) contains over one million biomedical concepts and five million concept names from more than 100 controlled vocabularies, including the Medical Subject Heading (MeSH), the thesaurus that is used for indexing MEDLINE citations. The Semantic Network (SN) represents a high-level abstraction from the UMLS Metathesaurus. The SN consists of 135 semantic types with 54 types of semantic relations (e.g., *IS-A *or *Part-of*) that relate the semantic types. Each UMLS concept is assigned one or more semantic types. For example, the MeSH term "Protein Tyrosine Phosphatase" is assigned the semantic types "Amino Acid, Peptide, or Protein Enzyme and Enzyme." In our study, we have linked the UMLS semantic types to the MeSH terms, and explored both as additional features for topic detection.

#### Evaluation metrics

For naïve Bayes classification, we report *accuracy*, the percentage of documents with the correct prediction. Accuracy in our study is equivalent to "precision", which is typically applied to evaluate information retrieval performance. To evaluate the method of group-wise-average hierarchical clustering, we have applied the TDT evaluation *cost of detection, C*_*D *_[10]*. C*_*D *_combines miss (*P*_*M*_) and false alarm (*P*_*FA*_) errors into a single number,

*C*_*D *_= *C*_M _* *P*_M _* *P*_*T *_+ *C*_FA _* *P*_FA _* (1-*P*_*T*_)     (3)

where *C*_*M *_and *C*_*FA *_are the costs of a miss and a false alarm, respectively (equal to 1). *P*_*T *_is a training data specific a priori target probability of a story discussing a topic,

PT,i=∑jA(i,j)∑i∑jA(i,j)     (4)
 MathType@MTEF@5@5@+=feaafiart1ev1aaatCvAUfKttLearuWrP9MDH5MBPbIqV92AaeXatLxBI9gBaebbnrfifHhDYfgasaacH8akY=wiFfYdH8Gipec8Eeeu0xXdbba9frFj0=OqFfea0dXdd9vqai=hGuQ8kuc9pgc9s8qqaq=dirpe0xb9q8qiLsFr0=vr0=vr0dc8meaabaqaciaacaGaaeqabaqabeGadaaakeaacqWGqbaudaWgaaWcbaGaemivaqLaeiilaWIaemyAaKgabeaakiabg2da9maalaaabaWaaabuaeaacqWGbbqqcqGGOaakcqWGPbqAcqGGSaalcqWGQbGAcqGGPaqkaSqaaiabdQgaQbqab0GaeyyeIuoaaOqaamaaqafabaWaaabuaeaacqWGbbqqcqGGOaakcqWGPbqAcqGGSaalcqWGQbGAcqGGPaqkaSqaaiabdQgaQbqab0GaeyyeIuoaaSqaaiabdMgaPbqab0GaeyyeIuoaaaGccaWLjaGaaCzcamaabmaabaGaeGinaqdacaGLOaGaayzkaaaaaa@4D8C@

PT=1N∑iPT,i     (5)
 MathType@MTEF@5@5@+=feaafiart1ev1aaatCvAUfKttLearuWrP9MDH5MBPbIqV92AaeXatLxBI9gBaebbnrfifHhDYfgasaacH8akY=wiFfYdH8Gipec8Eeeu0xXdbba9frFj0=OqFfea0dXdd9vqai=hGuQ8kuc9pgc9s8qqaq=dirpe0xb9q8qiLsFr0=vr0=vr0dc8meaabaqaciaacaGaaeqabaqabeGadaaakeaacqWGqbaudaWgaaWcbaGaemivaqfabeaakiabg2da9maalaaabaGaeGymaedabaGaemOta4eaamaaqafabaGaemiuaa1aaSbaaSqaaiabdsfaujabcYcaSiabdMgaPbqabaaabaGaemyAaKgabeqdcqGHris5aOGaaCzcaiaaxMaadaqadaqaaiabiwda1aGaayjkaiaawMcaaaaa@3E6A@

where A(*Ai, j*) is number of articles for topic *i*, and phenotype/gene *j*, *P*_*T*,*i *_is priori target probability for topic *i*, *N *represents number of topics in training data, and *P*_*T *_is the average priori target probability across all topics. *P*_*T *_is 0.038 and 0.071 for OMIM training data and the other annotated corpus respectively. The probability of missing *P*_*M *_is given by the number of documents in the gold-standard reference cluster, butt not present in the system cluster divided by the size of the reference cluster. The probability of false alarm *P*_*FA *_is given by the number of documents in the system cluster that are not present in the reference cluster. To get the final C_*D*_, we average the detection cost for each cluster over the topics (*topic-weighted-score*). The topic-weighted-score counts each document's contribution to the total cost equally. The disadvantage of this evaluation is that if a single document is missed in a small topic, the final cost can be affected dramatically.

#### Applying topic spotting and topic clustering to datasets

We have evaluated both topic spotting and topic clustering models with the two text collections we described earlier; namely, the OMIM database and the biological review articles. We have explored the combinations of word features in title and abstract. Additionally, we have explored the semantic features MeSH terms and the UMLS semantic concepts and types as additional features for topic detection.

For topic spotting, we have trained on a single naïve Bayes classifier with the "no_dup" documents (in Table 2). We performed ten-fold cross validation to assign each document to one of the 25 topics that are described in Table 2. The learning features include word features that appear in the title and abstract. We have also explored using the MeSH terms as additional learning features.

Since the OMIM topics are quite general, we hypothesize that general concepts may be useful features for the classification task. We have therefore also included the UMLS semantic types that have been assigned to the MeSH terms as the additional learning features. Furthermore, we have explored replacing each specific MeSH term with its general heading (i.e., the second level of MeSH terms in the hierarchy; e.g., replacing Phosphotyrosine with Amino Acids, Peptides, and Proteins) and applying the general headings for the training and the testing.

For topic clustering, we have deployed a slightly different approach. The OMIM database is organized by records, where each record represents a phenotype/gene. We first performed topic clustering for each OMIM record and then aggregated the clustering result; this approach is different from the approach of topic detection, in which the OMIM records are aggregated to form a giant collection of articles with the total of 25 topics. Processing per record represents the way topic clustering techniques will be applied in real world scenario as we would like to be able to cluster the articles into their respective topics given a dynamically selected set of articles. We have applied the similar topic clustering approaches to the additional set of biological review articles.

## Authors' contributions

ML has prepared the data from the OMIM and performed all the experiments under HY's guidance. WW has prepared manually annotated corpus for additional experiments.

## Figures and Tables

**Table 1:** Percentage of the accuracy (Acc.) when apply naïve Bayes (*NB*) to detect topics in OMIM with different learning features.

NB	S	S+M	S+M+T	S+M+T+A	M	T+A	GM	GM+M
Acc.	54.4	62.2	63.3	66.4	62.6	65.9	47.4	62.0

S = semantic sypes

M = MeSH terms

T = title

A = Abstract

GM = general MeSH terms

**Table 2:** OMIM topics and the number of documents that have been assigned to each topic.

Topics	Total Number	No_dup*	Topics	Total Number	No_dup*
CLONING	7719	2397	ANIMAL MODEL	879	261
MAPPING	7487	1760	CYTOGENETICS	770	222
MOLECULAR GENETICS	7139	2347	OTHER FEATUERS	349	90
CLINICAL FEATURES	6917	3044	HETEROGENEITY	298	62
GENE FUNCTION	6469	2960	HISTORY	131	42
GENE STRUCTURE	2444	165	EVOLUTION	126	16
INHERITANCE	1513	385	ALLELIC VARIANTS	122	80
DIAGNOSIS	1300	193	NOMENCLATURE	99	9
POPULATION GENETICS	1163	213	GENOTYPE	91	12
PATHOGENESIS	1062	216	GENE FAMILY	83	25
PHENOTYPE	1034	270	GENE THERAPY	51	21
BIOCHEMICAL FEATURES	993	314	GENETIC VARIABILITY	47	17
CLINICAL MANAGEMENT	974	284	**Total:**	**49,260**	**15,405**

* The number of references that have been assigned to only one topic, not others, within an OMIM entry.
